# Successful desensitization to horse antithymocyte globulin for aplastic anemia: two case reports and literature review

**DOI:** 10.1186/s40780-025-00421-w

**Published:** 2025-02-26

**Authors:** Satoshi Yuyama, Mitsuaki Oura, Tatsuya Isezaki, Daisuke Ikeda, Kanayuki Kitahara, Ryohkan Funakoshi, Kosei Matsue

**Affiliations:** 1 Department of Pharmacy, Kameda Medical Center, Kamogawa, Chiba Japan; 2 Division of Hematology/Oncology, Kameda Medical Center, Kamogawa, Chiba Japan; 3 Department of Hematology and Oncology, the University of Tokyo Hospital, Tokyo, Japan; 4 Department of Hematology and Oncology, Okayama University Hospital, Okayama, Japan

**Keywords:** Aplastic anemia, Antithymocyte globulin, Desensitization, Skin test

## Abstract

**Background:**

Horse antithymocyte globulin (hATG) is an important therapeutic option for aplastic anemia (AA). However, hATG carries the risk of fatal anaphylaxis, and skin tests are performed to identify high-risk patients. We report on the successful desensitization of two AA patients with positive skin tests to hATG.

**Case presentation:**

Case 1: A 72-year-old man with a history of successful treatment with rabbit ATG was referred for pancytopenia. Neutrophil, reticulocyte, and platelet counts were 546 /µL, 32,000 /µL, and 19,000 /µL, despite the oral administration of eltrombopag and cyclosporine. Bone marrow biopsy showed hypocellularity, and he was diagnosed with relapsed severe AA. Case 2: A 69-year-old man was referred for anemia and thrombocytopenia, and diagnosed with non-severe AA. Neutrophil, reticulocyte, and platelet counts were 2,044 /µL, 23,000 /µL, and 37,000 /µL. Bone marrow biopsy revealed hypocellularity. Neither patient had a history of allergy, and the skin prick test (SPT) of hATG was negative, but the intradermal test (IDT) was positive. The result of the IDT in case 2 was reproducible. They received hATG desensitization under close monitoring of vital signs in our high-care unit. The protocol consisted of gradually increasing doses of hATG (four intradermal, two subcutaneous, and four intravenous (IV) push) and some premedications prior to administration of the full dose IV drip. They completed the course without developing any systemic allergic reactions.

**Conclusions:**

Despite the risk of anaphylaxis, hATG desensitization can be beneficial in AA patients with a positive skin test, especially when no alternative is available or hATG is preferred.

## Background

Aplastic anemia (AA) is characterized by pancytopenia with hypocellular bone marrow (BM) in the absence of marrow fibrosis or the infiltration of malignancy. Antithymocyte globulin (ATG)-based immunosuppressive therapy (IST) is the standard treatment for severe AA (SAA) patients who are ineligible for transplantation, and non-severe AA (NSAA) patients who are transfusion dependent [1]. ATG is derived from either horse or rabbit, and horse ATG (hATG) has been reported to be equally or more effective than rabbit ATG (rATG) [2–6]. hATG has become available in Japan since 2023, providing a new treatment option for AA. hATG carries the risk of fatal anaphylaxis, and skin tests are performed to identify high-risk patients [7, 8]. Although desensitization has been attempted for high-risk patients in previous studies, to the best of our knowledge, there have been no reports from Japan. We report on the successful desensitization of two AA patients with positive skin tests to hATG.

## Case presentation

### Case1

72-year-old Japanese male who had the history of SAA 2 years prior was successfully treated with rATG and achieved full hematologic recovery. He had no history of allergy. He was referred for pancytopenia (WBC 1400 /µL, ANC 546 /µL, Hb 7.9 g/dL, PLT 19,000 /µL, reticulocyte count 32,000 /µL) and subcutaneous bleeding. Spinal MRI showed increased adipose tissue, and BM puncture revealed hypocellular marrow. Paroxysmal nocturnal hemoglobinuria (PNH) type blood cells were detected (PNH-R 0.003%, PNH-N 0.056%). He was diagnosed as relapsed SAA (severity criteria: stage 4), and a second IST using hATG was planned (hATG 40 mg/kg for 4 days). Skin tests were performed prior to hATG administration to evaluate the risk of anaphylaxis. Chlorpheniramine 10 mg was used as a premedication for platelet transfusion four days before the skin test, but no other antihistaminic drugs were used in the previous week, nor topical steroids were used during the previous three weeks. Skin prick test (SPT) with undiluted (50,000 µg/mL) hATG was negative. Intradermal test (IDT) using 0.02 mL of 1000-fold diluted (50 µg/mL) hATG showed a wheal with 8-mm diameter. This was more than 3 mm larger than that of the control (the same volume of saline showed a wheal with 3-mm diameter), so it was evaluated as positive. The administration of hATG was discontinued. However, cyclosporine and eltrombopag without ATG failed to resolve his transfusion dependency. hATG desensitization therapy was administered under close monitoring of vital signs in our high care unit, following the protocol of Millar and Wolanin et al. [9, 10] (Table 1). Acetaminophen 500 mg, chlorpheniramine 10 mg, famotidine 20 mg, and methylprednisolone 80 mg were administered as premedication before the start of desensitization and before the full dose. He completed four days of treatment without any local or systemic allergic reactions. Cyclosporine 40 mg/day and eltrombopag 100 mg/day, followed by darbepoetin alfa and G-CSF as supportive therapy, led to a partial response (PR).

**Table 1:**
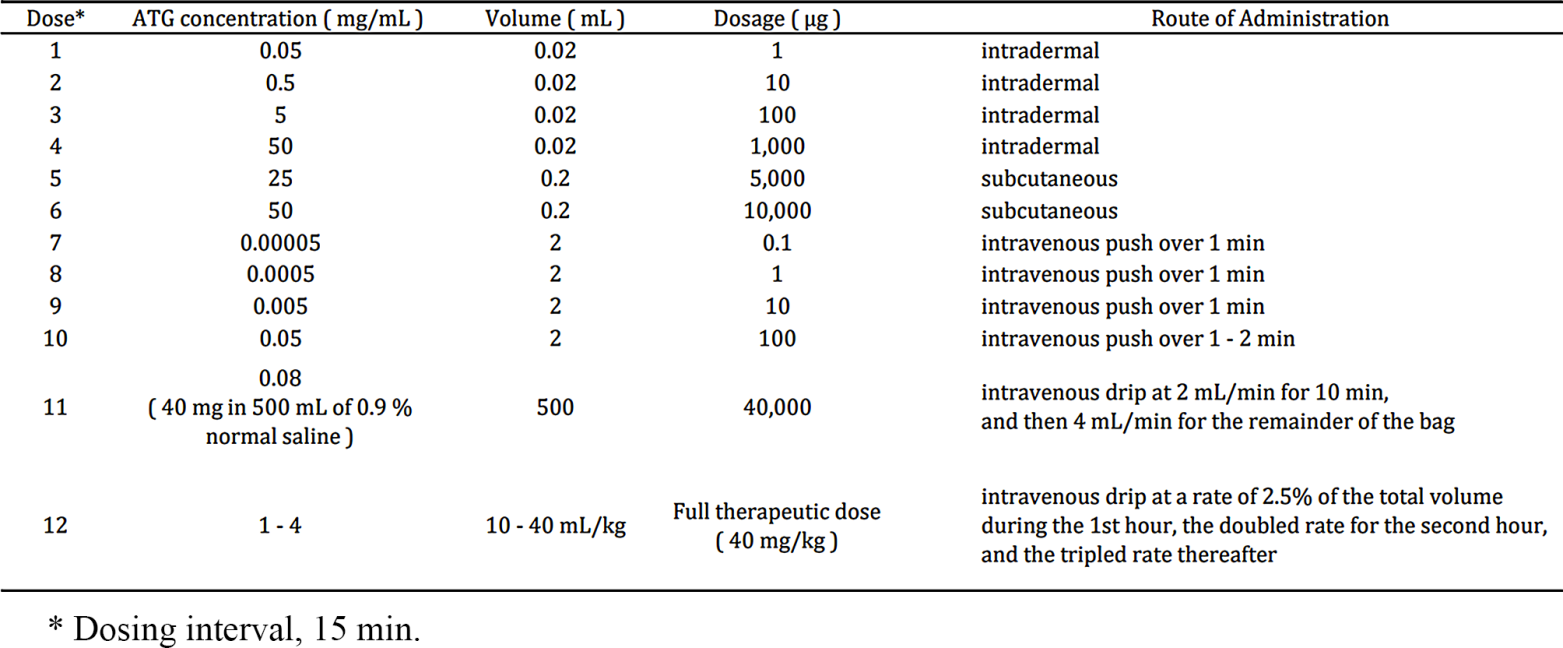
Desensitization protocol for hATG

### Case2

**Fig. 1:**
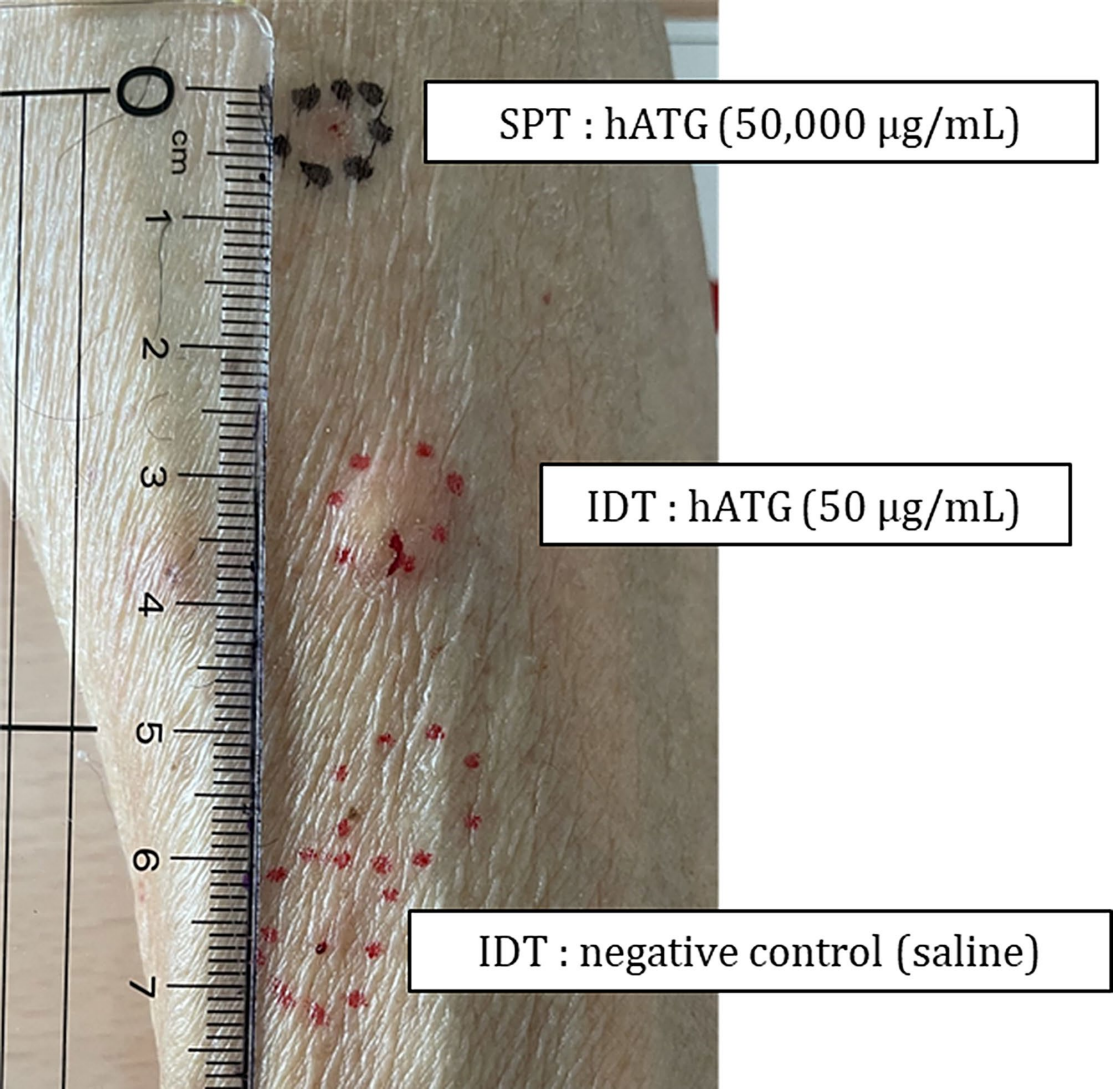
The results of the skin tests in case 2 are shown. Skin prick test (SPT) with undiluted (50,000 µg/mL) hATG was negative. Intradermal test (IDT) using 0.02 mL of 1000-fold diluted (50 µg/mL) hATG was positive because of a wheal which were at least 3 mm larger in diameter than the negative control (the same volume of saline). IDT was repeated contralaterally with similar results

A 69-year-old Japanese male with no history of allergy. He was referred for pancytopenia (WBC 2800 /µL, ANC 2044 /µL, Hb 6.7 g/dL, PLT 37,000 /µL, reticulocyte count 23,000 /µL). BM puncture revealed hypocellular marrow. PNH-type blood cells were detected (PNH-R 0.021%, PNH-N 0.043%). Because there were no agents that could have induced AA, he was diagnosed as NSAA (severity criteria: stage 3). Since he was transfusion dependent, the first IST with hATG (hATG 40 mg/kg for 4 days) was planned. To identify the risk of anaphylaxis, a skin test was performed prior to hATG administration with the same method as in case 1. No antihistamines had been used for 1 week, and no topical steroids had been used for more than 3 weeks. SPT was negative and IDT was positive because of a wheal with 10 mm-diameter, which was at least 3 mm larger in diameter than the control (the same volume of saline showed a flare with 4-mm diameter, Fig. 1). Considering the possibility of false positives, IDT was repeated contralaterally with similar findings. Desensitization therapy was administered in the same method as in Case 1. Although a wheal was observed as a local adverse reaction during the dose escalation phase, desensitization was continued as there were no changes in vital signs. He successfully completed four days of hATG treatment without any systemic allergic reaction. Subsequently, we obtained a PR.

### Discussion and conclusions

We successfully performed hATG desensitization to two high risk AA patients with SPT negative and IDT positive, following the protocol of Millar and Wolanin et al. [9, 10]. hATG rarely causes fatal anaphylaxis [11]. Skin testing is considered useful in identifying patients at high risk of anaphylaxis, and the U.S. Food and Drug Administration (FDA) strongly recommends skin testing prior to administration [7]. On the other hand, the British Society of Hematology (BSH) recommends intravenous infusion test administration instead of skin tests because of the high incidence of false-positive and false-negative results [1]. In Japan, both skin tests and intravenous infusion tests are available, but if a skin test is positive, a change in the treatment plan should be considered on the risk-benefit balance [8]. Given that elderly patients are a risk factor for severe anaphylaxis [12], we chose skin testing to identify risk. Although strategies when positive skin tests for hATG are have not been established, desensitization is one therapeutic approach for immediate IgE-mediated drug hypersensitivity reactions such as anaphylaxis [13].

In a literature review, hATG desensitization was performed in 15 patients (Table 2). IDT was performed in all patients before administration and was positive in all cases [9–11, 14–17]. SPT was performed before administration in 11 patients, and one patient was positive. Thirteen patients were successfully desensitized. Of the 13 patients who successfully desensitized, six patients had symptoms of wheals, fever, headache, and arthralgia during desensitization but were able to complete the administration. In the two cases where desensitization failed, anaphylaxis was observed in both cases. SPT was not performed in the two cases with anaphylaxis and in the two cases with wheals and urticaria during desensitization. It has been reported that a case of death due to anaphylaxis was positive for SPT [11].

**Table 2:**
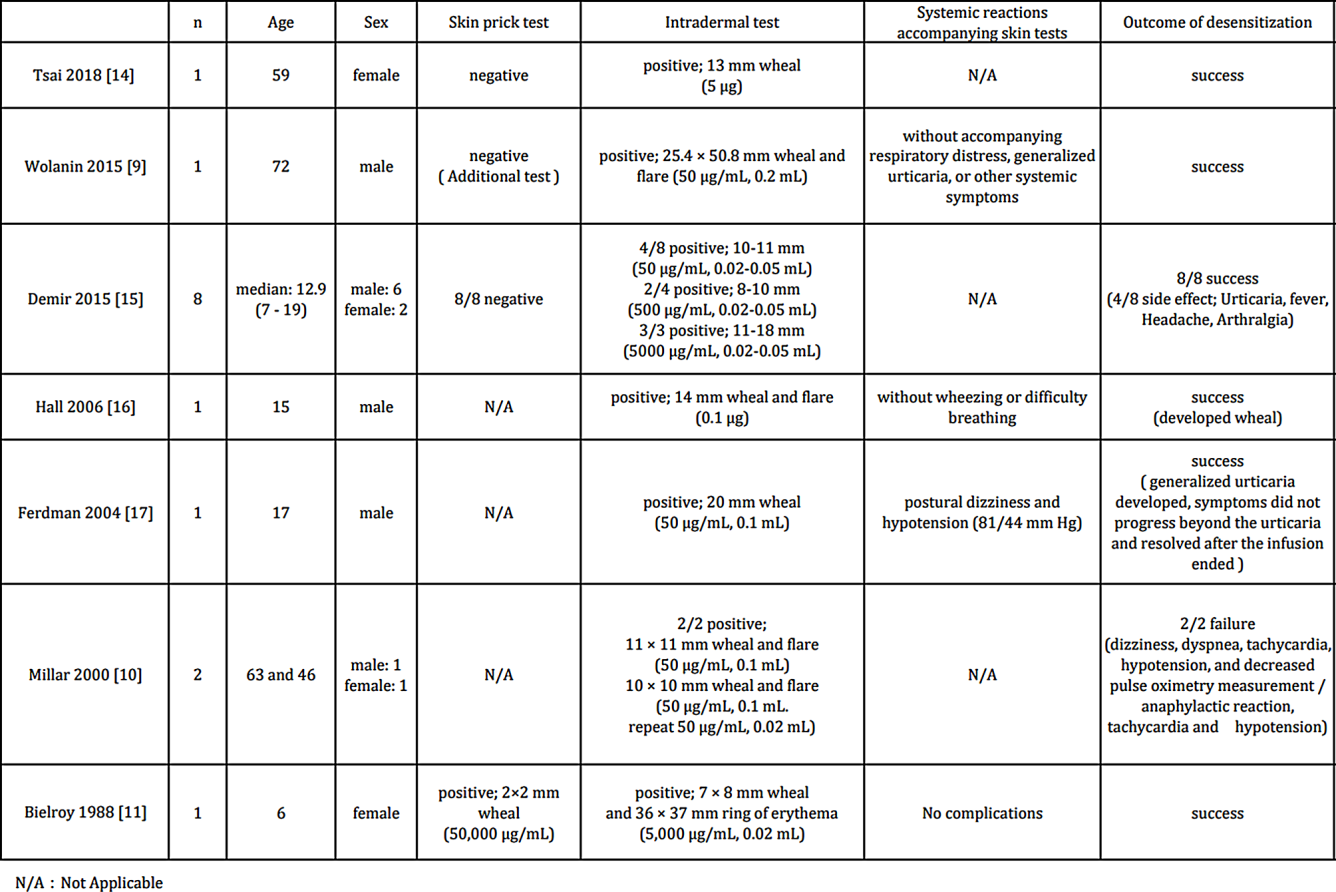
Literature review on desensitization to hATG

SPT is the most reliable and cost-effective tool for the diagnosis of type I immediate-type IgE-mediated allergy, being safe, high in sensitivity and specificity, and used in the initial approach. IDT should be considered in patients with a strong clinical suspicion of immediate IgE-mediated allergy and a negative SPT. Compared with SPT, IDT has a lower positive predictive value because of its increased sensitivity but lower specificity. Factors such as infants and the elderly, and the use of antihistaminic drugs can cause false negative results [18].

Both cases had a negative SPT and a positive IDT. Although it is possible that the IDT results were false-positives, we performed desensitization because we could not rule out a potential risk of anaphylaxis. Wolanin et al. emphasized the importance of SPT and suggested that patients who are SPT negative and IDT positive may tolerate desensitization [9]. Our results support this opinion.

While it is an option for allogeneic hematopoietic stem cell transplant (HSCT), long-term survival rates are only about 70% in patients over 40 years of age due to comorbidity and treatment toxicity, even with allogeneic HSCT from HLA-matched sibling donors (MSD) [19]. The BSH guidelines recommend IST as the first-line treatment for elderly AA patients, and allogeneic HSCT should be considered for those who do not respond to first-line IST [1]. Another issue for elderly AA patients is the limited availability of MSD. In light of these facts, the indication for allogeneic HSCT should be carefully considered. In our cases, the patients were ineligible for allogeneic HSCT due to age and lack of MSD.

Although there is a risk of anaphylaxis, in cases where no alternative is available or where hATG is preferred, hATG desensitization can be beneficial for AA patients who are SPT negative and IDT positive, under close monitoring of vital signs.

## Data Availability

All data generated or analyzed during this study are included in this published article.

## Abbreviations

hATG: Horse antithymocyte globulin
AA: Aplastic anemia
SPT: Skin prick test
IDT: Intradermal test
IV: Intravenous
BM: Bone marrow
ATG: Antithymocyte globulin
IST: Immunosuppressive therapy
SAA: Severe aplastic anemia
NSAA: Non-severe aplastic anemia
rATG: Rabbit antithymocyte globulin
PNH: Paroxysmal nocturnal hemoglobinuria
PR: Partial response
FDA: Food and Drug Administration
BSH: British Society of Hematology
HSCT: Hematopoietic stem cell transplant
MSD: Matched sibling donors
